# Fabricating the Superhydrophobic Nickel and Improving Its Antifriction Performance by the Laser Surface Texturing

**DOI:** 10.3390/ma12071155

**Published:** 2019-04-10

**Authors:** Junyuan Huang, Songbo Wei, Lixin Zhang, Yingying Yang, Song Yang, Zejun Shen

**Affiliations:** 1 Department of Petroleum Equipment, PetroChina Research Institute of Petroleum Exploration & Development, Beijing 100083, China; jyhuang2016@163.com (J.H.); weisongbo@petrochina.com.cn (S.W.); judyzhang@petrochina.com.cn (L.Z.); 2 Institute of Semiconductors, Chinese Academy of Sciences, Beijing 100083, China; yangsong@semi.ac.cn

**Keywords:** laser surface texturing, nickel, superhydrophobic surface, water lubrication, antifriction performance

## Abstract

The superhydrophobic surface can change the friction property of the material, reduce the adhesion of the friction interface, and produce a certain slip, thereby reducing the friction coefficient. The laser has high energy, high density, and is especially suitable for the surface treatment of materials. The laser surface texturing is a good way to construct superhydrophobic surfaces. The experiment uses a nanosecond pulse laser to construct the groove texture on the nickel surface. The contact area between the air and the droplets retained on the rough surface is increased, effectively preventing the water droplets from entering the gully of the surface microstructure, reducing the water droplets and the solid surface. The contact area ultimately makes the surface exhibit excellent superhydrophobicity. A superhydrophobic nickel surface having an apparent contact angle of water (ACAW) of 160° and a sliding angle (SA) of less than 10° was prepared. The MM-W1B vertical universal friction and wear tester was used to test the groove texture samples with different depths. The surface texture can capture the wear debris generated by the wear and store the lubricant, which is beneficial to the formation of fluid dynamic pressure lubrication and improve the load. The friction coefficient is reduced from 0.65 of the unprocessed surfaces to 0.25 after the texturing, and the friction performance is greatly improved.

## 1. Introduction

The study of the superhydrophobic surface in bionics is one of the hotspots today. The preparation of mechanical parts into super-hydrophobic surfaces will greatly reduce the friction between parts, reduce wear, and prolong the service life, and it will also reduce the cost of parts, which has great economic benefits [1,2]. Many experts and scholars conduct research to improve the performance of materials. Garcia [3] obtained surface layers with an increased hardness from 172 HV to 1001 HV and 305 HV respectively by using the nitrogen and carbon-based gasses in the nitrided stainless steel plates alloying process, at the same time to functionalize it to obtain superhydrophobic properties. Mozammel [4] increased the roughness of the 316L stainless steel by coating the TiO_2_ on substrates via sol–gel/dip coating method, made the apparent contact angle of water increased from 142.5° to 168.5°. Metal surfaces with special wetting properties (hydrophilic/hydrophobic) have important application value in anti-corrosion [5], drag reduction [6], self-cleaning [7], and anti-icing [8,9,10,11]. Elisa [12] studied and found that the superhydrophobic coatings reduce the total friction losses of the mini-turbogenertor by similar to 7%, while the smooth coatings reduce them by similar to 5%. Research on changing and regulation of the wetting properties of metal surfaces is of great significance for broadening its application.

The laser surface texturing operation process is simple, non-toxic, non-polluting, high stability, and can handle a wide variety of materials, which has great advantages in superhydrophobic surface preparation [13,14]. In the research that has been carried out, most of the work uses picosecond and femtosecond lasers [15,16,17,18]. However, the research on surface texture of nanosecond pulse laser is relatively small, but it is simple and efficient [19,20]. It is necessary to conduct a detailed study of its preparation process to prepare super-hydrophobic surfaces.

The nickel can improve the mechanical properties of steel, increase the strength, toughness, heat resistance, the corrosion resistance, acid resistance, and magnetic permeability. Therefore, nickel-plated surface treatment is usually used to improve the surface properties of the material [21,22]. However, the wear resistance of the pure nickel layer is much lower than that of the nickel-based multiple plating layer [23]. Hadipour [24] studied that the wear resistance of the nickel coatings that were produced by magnet stirrer process at 2 A/dm^2^ and rotating disk cathode process at 4 A/dm^2^ were lower than the other coatings. Therefore, it is necessary to treat the pure nickel layer to improve its surface properties.

This paper aims to improve the friction properties of the pure nickel and improve the abrasion resistance of the pure nickel coating. Superhydrophobic films were prepared on the surface of nickel by nanosecond pulsed laser. The preparation process was studied and the wetting properties of superhydrophobic films and the tribological properties under water lubrication conditions were studied.

## 2. Experimental Section

In order to improve the antifriction performance of the nickel coating, the super-hydrophobic nickels were prepared first, and then their antifriction performance were studied. Follow the steps below:

### 2.1. Material Pretreatment

The purity of the nickel samples is 99.5%, which were polished to mirror gloss by 800 grit, 1000 grit, and 1500 grit sandpaper successively to ensure that the laser absorption rate of the surface is less than 10%. Then ultrasonically treated with the acetone, the absolute ethanol and the deionized water respectively for 10 min. Finally, it is blown dry with compressed air to remove impurities from the surface.

### 2.2. Laser Surface Texturing

**Figure 1:**
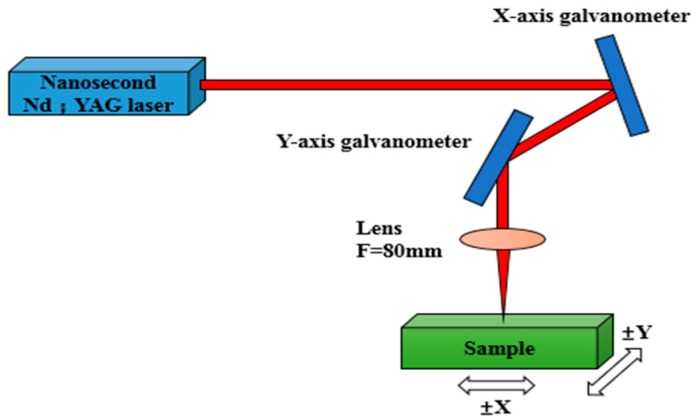
The nanosecond laser surface texturing system.

The as-prepared samples were irradiated with an optical fiber nanosecond pulse laser (IPG Photonics, YLPN-1-4×200-30-m, Oxford, MA, USA), with the polarized center wavelength of 1064 nm, the pulse duration about 100 ns, the repetition rate of 20 kHz, and the output beam quality factor of M^2^ < 1.5. The movement of the laser in the plane is controlled by a scanning galvanometer with an 80 mm focal lens and the focused spot size φ on the surface was 40 μm, as shown in Figure 1.

**Figure 2:**
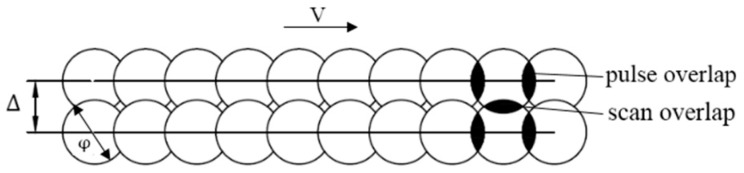
The scheme of the laser surface texturing processing.

The laser surface texturing is carried out in the air with the processing path crossed horizontally and vertically. As shown in Figure 2, the pulsed laser energy density $e_{p}$ on the surface of the material is defined in Equation (1), depending on the total energy received by the surface of the processed material $E_{p}$, the surface area S, and the unitless overlapping rate of α which is defined in Equation (2):

(1) $$e_{p}=\frac{E_{p}}{\alpha S},$$

and

(2) $$\alpha =\frac{∆}{\varphi }\;,$$

For example, if the area between the two lines is processed in one direction, the total energy received by the surface of the machined material is a function of the length L of the area, the processing speed V, and the laser power P:

(3) $$E_{p}=\frac{L}{V}\times P,$$

The area of the calculated area:

(4) S=L×φ,

So, Equation (1) can be simplified as

(5) $$e_{p}=\frac{P}{∆\;\times \;V}\;,$$

By changing the interval ∆, the speed V and the laser power P, a series of different energy densities are obtained.

To ensure the laser parameters controllable, the interval ∆ = 0.1 mm and the laser power *P* = 20 W is numerically fixed, only change the speed *V* to make the energy density *e_p_* changeable. In this experiment, the energy density range was set from 0.05 to 200 J/cm^2^.

### 2.3. Post-Treatment of Materials

After the laser processing, they were ultrasonically treated with the acetone, absolute ethanol, deionized water for 10 min, and dried with compressed air to remove surface impurities and oil stains, and then left them in the air for two weeks.

### 2.4. The Measurement of the Wettability

The apparent contact angle of water (ACAW) and sliding angle (SA) of the surfaces were measured by the contact angle measuring instrument (OCA20, DataPhysics, Filderstadt, Germany). The sample was placed on an inclined surface having an angle of 10°, and if the water droplet dripped quickly after falling on the surface, the surface SA was considered to be less than 10°. The amount of the water droplets was 2 μL and the average value of the three different positions was taken. According to the measured results, the relationship between the laser energy density and the wettability can be studied, as well as the judgement of whether the superhydrophobic surface was prepared.

### 2.5. The Antifriction Performance

**Figure 3:**
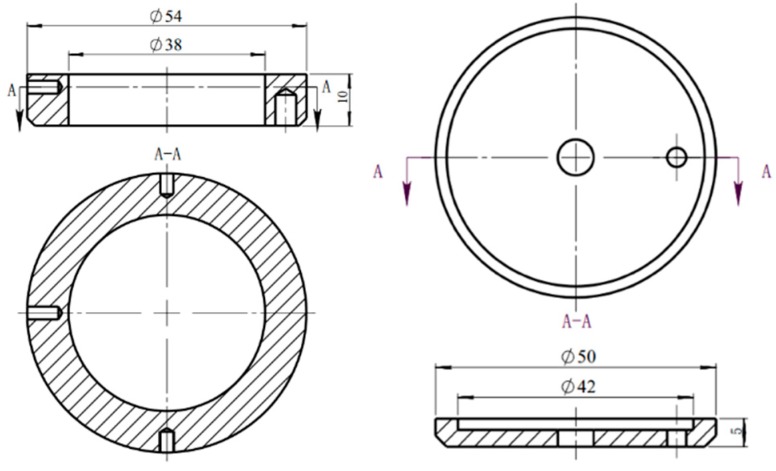
The size of the experimental materials.

The MM-W1B vertical universal friction and wear tester (Beijing Times Technology, Beijing, China) was used to carry out the experiment under the condition of the normal temperature and the water lubrication. The disc-plate grinding method was adopted and the empirical parameters of the actual working condition of the plunger in the petroleum machinery were selected. The test piece did not move during the experiment, and the upper test piece was rotated, and the rotation speed was 400 r/min with the load of 10 N, the friction time of 20 min, and each group of the experiments was repeated for 3 times. The friction coefficient was directly derived from the computer during the experiment. The shape and dimensions of the test pieces are shown in Figure 3. The scanning electron microscopy (TESCAN LYRA3 FEG-SEM/FIB, Brno, Czech Republic) and the white light interferometer (ZYGONexView, ZYGO, Middlefield, CT, USA) were used to observe the two-dimensional and three-dimensional morphology of the sample surfaces, and the rough structures formed on the surfaces were observed.

## 3. Result and Discussion

**Figure 4:**
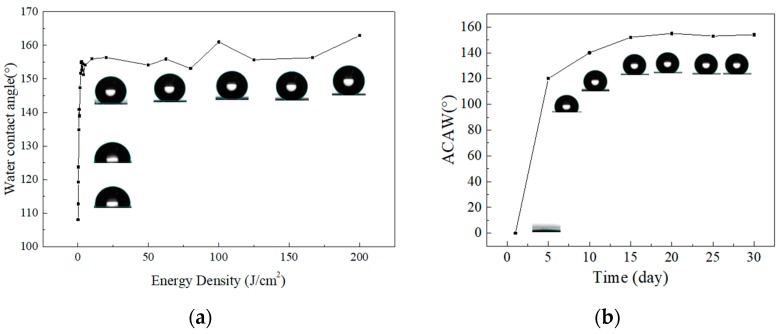
(**a**) The variation of the A apparent contact angle of water (ACAW) of the nickel surfaces with the laser energy density; (**b**) the apparent contact changes over time.

In order to study the relationship between the energy density of the laser and the wetting property of the nickel, the energy density range is set from 0.05 to 200 J/cm^2^. The ACAW is measured as shown in Figure 4a. Within a certain energy density range, the ACAW increases linearly with the energy density, and finally tends to be stable. When the energy density is less than 2 J/cm^2^, the ACAW tends to increase linearly with the increase of energy density. When the energy density is greater than 2 J/cm^2^, the ACAW exceeds 150°. When measuring the SA, the water droplets can quickly slide off the inclined surface of 10°. It can be seen that when the energy density exceeds 2 J/cm^2^, the surface exhibits superhydrophobicity. Processing the groove texture on the nickel surface does change the surface wetting properties, resulting in a superhydrophobic nickel surface.

Actually, the nickel surface studied exhibited superhydrophilicity immediately after being irradiated. However, the ACAWs on the surface increased over time, eventually becoming large enough to classify the surface as superhydrophobic. The storage conditions significantly affected this process. When the samples were stored in CO_2_, O_2_, and N_2_ atmospheres, the wettability transition was restrained. The transition was accelerated in atmosphere that was rich with organic compounds [25]. This wettability transition process was mainly caused by the adsorption of organic compounds from the surrounding atmosphere onto the oxide surface [26]. Figure 4b shows the variation of apparent contact over time. It can be seen that the prepared superhydrophobic surface has stability during the experimental time.

**Figure 5:**
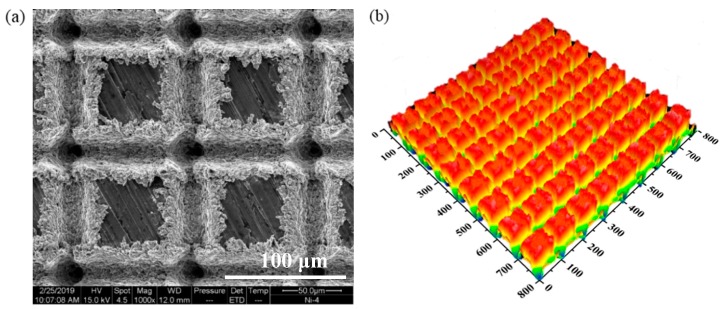
The surface morphology of the textured nickel (**a**) two-dimensional (**b**) three-dimensional.

In order to observe the morphology of the groove texture of the sample after the surface texturing, a sample having an energy density of 4 J/cm^2^ was taken as an example. The scanning electron microscopy (SEM) was used to observe the surface morphology of the samples. It was found that the surface has many micro-nano structures, as shown in Figure 5a. The three-dimensional topography of the nickel after laser texturing was measured by a white light interferometer, as shown in Figure 5b. After the groove texture processed, the surface is crisscrossed, and the surface is rough after the laser processing. The contact area of the air trapped on the surface increases with the droplets, effectively preventing the water droplets from entering the gullies of the surface microstructure. A large amount of air is trapped in the rough structure, so the water droplets encountered the air, thereby reducing the contact area between the water droplets and the solid surface, and finally the surface exhibits excellent super-hydrophobicity [27,28,29,30,31].

**Figure 6:**
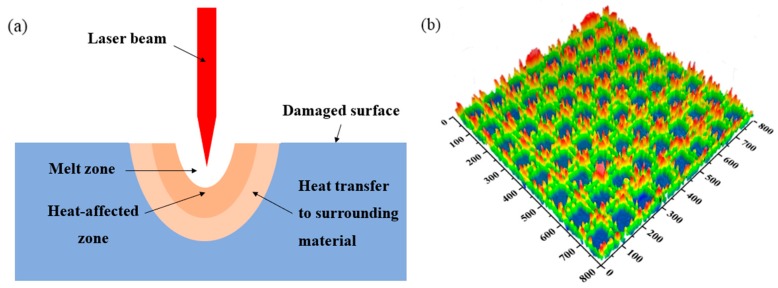
(**a**) The schematic diagram of the laser surface texturing; (**b**) The three-dimensional topography of the groove of nickel with an energy density of 100 J/cm^2.^

The sample forms regular grooves and micro-nanostructures due to the illumination of the laser. As shown in Figure 6a. The material in the illumination area is burned and removed, and the energy is concentrated at the position where the laser light is superimposed, so that the amount of material removal is increased, thereby generating a regularly arranged tens of micrometer-depth grooves. In the area where the laser irradiation is relatively small, the surface temperature of the molten material is rapidly lowered due to the movement of the laser beam, and re-solidified in the non-irradiated area to form a solidified protrusion [10,11]. Adhesives of various shapes, the columnar, the spherical, and the disc-shaped protrusions are attached to these protrusions, ranging in size from several micrometers to ten micrometers. The formation of these grooves and protrusions increases the surface roughness of the nickel, thereby affecting the wetting properties [32,33], as shown in Figure 5b. However, as shown in Figure 6b, when the energy density is greater than 100 J/cm^2^, the surface does not form a significant groove and has many protrusions, the metal melts too much and fills the grooves, so that there are no obvious grooves on the surface, but protrusions formed by molten metal [34].

**Figure 7:**
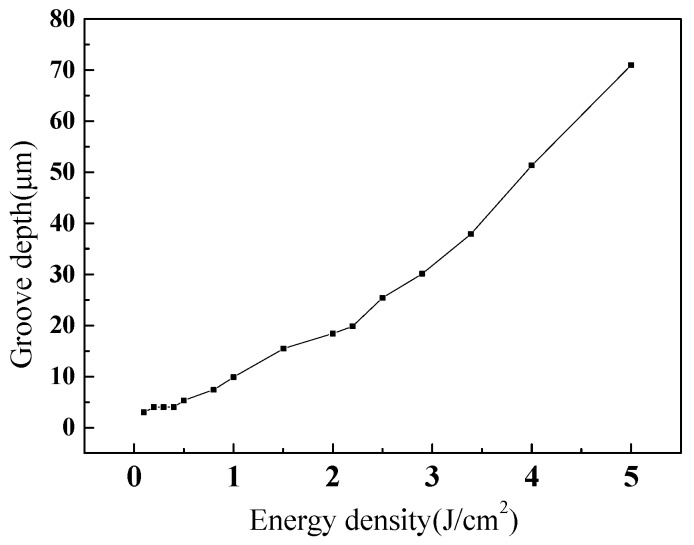
The depth of groove texture of nickel at different energy densities.

As the energy density increases, the depth of the trench gradually increases, taking the trench depth of nickel at different energy densities as an example, as shown in Figure 7.

**Figure 8:**
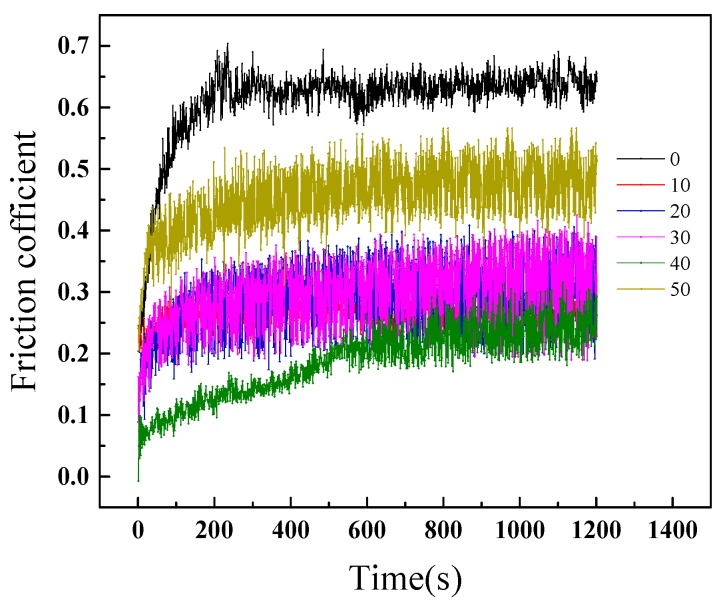
The friction coefficient of the superhydrophobic nickel surfaces with different depth groove texture.

In order to study the influence of different groove depths on the friction coefficient, the texture samples with texture depths of 10, 20, 30, 40, and 50 μm were selected for friction experiments with the load of 10 N, the speed of 400 r/min and the time of 20 min. The friction coefficients measured under the condition of room temperature with the water lubrication are shown in Figure 8. The surface friction coefficient of the groove texture is lower than that of the unprocessed smooth nickel surface, it is reduced from 0.65 to 0.25. The influence of the groove depth on the friction coefficient is nonlinear because the increase in the groove depth does not reduce the friction coefficient.

**Figure 9:**
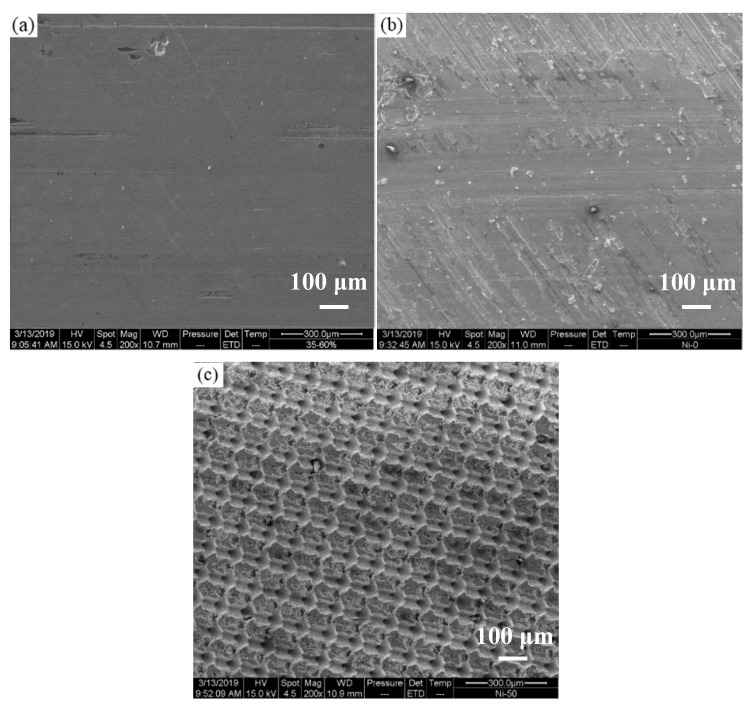
The topographical view of the groove texture of the nickel surface. (**a**) the surface of the as prepared nickel; (**b**) the surface friction morphology of the un-textured nickel; (**c**) the surface friction morphology of the textured nickel with the depth of 50 μm.

In order to observe the surface topography of the surface textured sample after the friction test, the surface friction morphologies of the test piece were observed by the SEM. Figure 9a shows the surface of the as prepared nickel. Figure 9b shows the surface friction morphology of the un-textured nickel. Figure 9c shows the surface friction morphology of the textured nickel with the depth of 50 μm. The un-textured surface of the friction test piece has obvious rolling marks and has severe adhesive wear, while the surface of the nickel with grooved texture is not obvious. When the groove depth is 50 μm, the rolling condition on the surface of the test piece is improved and the degree of adhesive wear is reduced. The surface is only slightly worn, and the morphology does not change significantly, which is consistent with the decrease of the friction coefficient [35].

The laser prepared surface groove texture formed a superhydrophobic surface, which really changed the friction coefficient of the nickel. The surface roughness is increased, and the wear mechanism of the surface of the test piece is mainly changed from adhesive wear to abrasive wear, which is not conducive to alleviating the wear of the friction pair and making the surface not wearable [36,37]. However, the super-hydrophobic property of the surface reduces the adhesion of the friction interface, and the water lubrication causes a certain slip between the friction interfaces, so the adhesion between the friction pairs is greatly reduced. The surface texture increases the contact area of the liquid and liquid, and the actual contact area of the solid is reduced, thereby reducing the friction. The groove texture can form a function of storing lubricant [19,20,32,33]. When the water film on the surface is thinned and destroyed, the water in the groove can be supplemented, which produces a secondary lubrication effect, and the metal surface always exists in the friction process [34,35]. The thickness of the continuous water film is conducive to the formation of fluid dynamic pressure lubrication, improve the load bearing capacity, reduce the frictional resistance, thus avoiding the occurrence of boundary lubrication or even dry friction. The laser interacts with the metal to increase the hardness of the surface and increase the wear resistance [38].

## 4. Conclusions

1. The surface texturing increases the roughness of the metal, and the contact area between the air and the droplets retained by the rough surface increases, effectively preventing the water droplets from entering the gully of the surface microstructure, and a large amount of air is trapped in the rough structure. The contact area between the water droplets and the solid surface is reduced, and a superhydrophobic nickel surface having an ACAW of more than 160° is finally obtained.

2. The texture can also capture the wear debris generated by the wear, reduce the furrow effect and prevent the occurrence of severe wear.

3. The texture can form a function of storing lubricant. When the water film on the surface is thinned and destroyed, the water in the groove can be supplemented, which is favorable for forming hydrodynamic lubrication, improving load bearing capacity and reducing frictional resistance, avoiding boundary lubrication or even dry friction.

4. The superhydrophobic property of the surface reduces the adhesion of the friction interface. The water lubrication causes a certain slip between the friction interfaces, and the adhesion between the friction pairs is greatly reduced, further reducing the friction coefficient.
